# The Role of Sleep Effort as a Mediator Between Anxiety and Depression

**DOI:** 10.1177/00332941221149181

**Published:** 2023-01-03

**Authors:** Cristina Borges, Jason G. Ellis, Daniel Ruivo Marques

**Affiliations:** Department of Education and Psychology, 56062 University of Aveiro, Aveiro, Portugal; Northumbria Sleep Research Laboratory, Faculty of Health and Life Sciences, 5995 Northumbria University, Newcastle, UK; Department of Education and Psychology, 56062 University of Aveiro, Aveiro, Portugal; CINEICC - Center for Research in Neuropsychology and Cognitive Behavioral Intervention, Faculty of Psychology and Educational Sciences, University of Coimbra, Portugal

**Keywords:** Sleep effort, depression, anxiety, insomnia, higher education students, mediation analysis

## Abstract

Depression, anxiety, and insomnia are all conditions that share a complex bidirectional relationship. Sleep effort is a construct with cognitive and behavioral components that perpetuates insomnia. Although many studies have examined the associations between these three variables, no studies have yet examined sleep effort as a mediating variable between anxiety and depression and vice versa. Online versions of the Hospital Anxiety and Depression Scale and the Glasgow Sleep Effort Scale were administered to a sample of 1927 higher education students aged 18–40 years (75.9% women and 76% from 18 to 23 years old). As part of the survey, participants also completed a sociodemographic questionnaire. Mediation analysis indicated that sleep effort mediates the relationship between depression and anxiety, when the former was the predictor and the latter was the criterion. Moreover, sleep effort also mediated the relationship between anxiety and depression when the former was the predictor and the latter was the criterion, albeit in a lesser extent. Sleep effort appears to play a bidirectional mediational role between depression and anxiety, being a potential target for intervention.

## Introduction

Insomnia is the most common sleep disorder (Buysse & Harvey, 2017) with a prevalence of 3–22%, varying by the classification system used (Wilson & Attarian, 2017). It is characterized as the difficulty in initiating or maintaining sleep, waking up earlier than desired for three or more nights in a week, for at least 3 months, and causing significant impairment in daily functioning (APA, 2014; American Academy of Sleep Medicine, 2013; Marques et al., 2020). Individuals with insomnia experience fatigue, malaise, difficulty paying attention, concentration or memory difficulties, irritability, mood disturbances, daytime sleepiness, behavioral disorders, among other problems (Ong & Gehrman, 2017). Insomnia can be observed in isolation or co-morbidly with a chronic illness, such as panic disorder, depression, anxiety, personality disorders, adjustment disorders, and somatoform disorders (Lichstein et al., 2017; Marques et al., 2020; Perlis et al., 2017).

There are strong associations between insomnia and poor health outcomes such as quality of life, mood, sleepiness, cognitive and subjective functioning (Walsh, 2004). For this reason, the long-term health consequences of insomnia are significant and can affect the individual’s typical functioning and productivity, in addition to having high associated healthcare costs (Broomfield et al., 2005; Buysse & Harvey, 2017; Harvey, 2002). Symptoms of insomnia have been on the rise for decades. Between 2002 and 2012, it increased by 1.5%, as reported by Garland et al. (2018). Furthermore, with this progressive increase, insomnia-related costs have risen in the European population, for example (Riemann et al., 2017; Wittchen et al., 2011).

Over the years several models of insomnia have been developed, such as Espie et al.’s (2006) psychobiological inhibition model - based on the A-I-E (Attention, Intention, Effort) pathway, to understand insomnia. Related to this, is the preliminary model of sleep effort developed by Broomfield and Espie (2005) that begins with a period of acute insomnia which activates dysfunctional beliefs about sleep, potentially leading to chronic insomnia. The authors propose that sleep effort encompasses (i) anxiety about sleep and its consequences, generating anticipatory anxiety; and (ii) sleep control and performance effort, leading to sleep avoidance. A study by Hertenstein et al. (2015) concluded that greater sleep effort was associated with increased insomnia severity. This is congruent with the study by Baglioni et al. (2014) that found the need for perceived control regarding sleep was associated with more avoidance and safety behaviors, further underpinning the strong association between sleep effort and insomnia severity. As such, sleep effort is not only a construct with several cognitive and behavioral components, but can also serve as a perpetuating and maintenance factor in insomnia disorder (Broomfield & Espie, 2005).

Anxiety and depression are very common mental disorders that also have a significant impact on public health (Penninx et al., 2011). They are also frequently comorbid with each other (Asarnow & Manber, 2019; Baglioni et al., 2011; Buysse & Harvey, 2017).

Major depressive disorder is characterized by depressed mood or lack of interest in activities that were once pleasurable. This mood disturbance should be present almost every day and associated with feelings of sadness, hopelessness, and/or emptiness. Loss or increase of weight and/or appetite can also be a diagnostic feature, as well as insomnia or hypersomnia, psychomotor agitation or retardation, guilt, reduced attention and concentration, thoughts about death and/or suicide, fatigue, and lack of energy. Depression has significant implications for quality of life (APA, 2014; Malhi et al., 2014; Malhi & Mann, 2018). Compared to depression, there are several anxiety disorders, differing according to the situation or object that generates the irrational fear, anxiety, cognitive ideations or avoidance behavior. They must have also persisted for at least 6 months. The proportion of fear or anxiety is evaluated considering the context, culture, and individual characteristics (APA, 2014).

The literature indicates that anxiety is an important risk factor for depression (Baglioni et al., 2011; Breslau et al., 1996; Gregory et al., 2009; Roane & Taylor, 2008; Tiller, 2012), and individuals diagnosed with an anxiety disorder may also be diagnosed with depression. Although this relationship is bidirectional (Alvaro et al., 2013; Baglioni et al., 2010; Baglioni et al., 2011; Dolsen et al., 2014; Jacobson & Newman, 2017; Jansson-Fröjmark & Lindblom, 2008; Kalin, 2020; Sivertsen et al., 2012; Tiller, 2012), anxiety is more likely to trigger depression than vice versa (Cummings et al., 2014; Jacobson & Newman, 2017; Kaufman & Charney, 2000; Kessler & Wang, 2008; Starr et al., 2014). Research has also shown that sleep disturbances play a major role in both anxiety and depression (Baglioni et al., 2011).

Depression and anxiety are closely related and often comorbid conditions. In general, anxiety precedes depression, but the inverse relationship does also occur, considering that depression can develop without prior anxiety (Lavigne et al., 2015). Moffitt et al. (2009) found that 32% of their sample experienced major depression before developing anxiety. This study suggests depression precedes anxiety, based on the authors' findings.

Insomnia is associated with depressive episodes (Morgan & Clarke, 1997; Weissman et al., 1997) and anxiety disorders (Costa e Silva et al., 1996; Morin et al., 2006; Roth et al., 2006; Zhang et al., 2012). Albeit insomnia is often associated with anxiety and depression, it seems to be more strongly related to anxiety than depression (Taylor et al., 2005) with anxious individuals tending to develop sleep problems (Li et al., 2018), and consequently developing depression (Brown et al., 2001).

Sleep problems are very frequent in college students (Becker et al., 2018; Prichard et al., 2020; Silva et al., 2021). Taylor et al. (2011) report that college students constitute a commonly studied population when investigating the relationship between insomnia and mental health. According to a study by Gellis et al. (2014), this population present a strong association between sleep problems and unhealthy sleep behaviors, such as irregular sleep schedules, poor sleep environment, and arousal-promoting behaviors before bedtime, negatively affecting their health and well-being. Over 30% of college students have difficulties getting sufficient sleep and half report daytime sleepiness (Brown et al., 2006; Lund et al., 2010). Moreover, 30–50% of college students suffer from insomnia (Kloss et al., 2011; Manzar et al., 2015; Petrov et al., 2014), and they are more likely to develop depression and anxiety than the general population (Booth et al., 2015; Ibrahim et al., 2013). This is in line with the findings of Manzar et al. (2020) who concluded that insomnia, in addition to being common in young individuals under 25 years, is often accompanied by high stress levels and poor sleep habits. As such, the pertinence of studying this population might aid in terms of developing sleep health related prevention programs.

Thus, the purpose of this study is to investigate whether sleep effort constitutes a mediator between depression and anxiety and vice versa. Based on previous research we hypothesized that (1) sleep effort is associated with depression and anxiety; (2) sleep effort partially mediates the association between depression and anxiety, and (3) sleep effort partially mediates the association between anxiety and depression.

## Methods

### Design

This study comprises a cross-sectional design in which data was collected from a specific population (college students) and aims to make inference about the relationship of sleep effort on anxiety and depression. Participants were instructed to complete an online questionnaire. This questionnaire featured sociodemographic questions, and some scales such as the Hospital and Anxiety Depression Scale and the Glasgow Sleep Effort Scale (GSES).

### Participants

The sample from this study included 1927 participants, predominately women (75.9%), aged between 18 and 23 years = 76%, 24 and 30 years = 17.2%, 31 and 40 years = 6.8%. Pertaining to marital status, 91.7% were single, 5% were married and 3.3% were in ‘another’ marital situation. Regarding academic degree, 69.5% were undergraduates, 25.1% were studying at postgraduate level, and 5.4% were studying at PhD level. In terms of subject areas, the sample comprised predominately social sciences and humanities (28.1%), science and engineering (25.6%), arts (13.1%), health and biomedical sciences (13%), behavioral sciences (10.2%), languages and literature (8.1%), among others (1.9%). Regarding student status, most of the sample studied on a full-time basis (80.6%), with the remaining reporting being working students (16.6%) or “others” (2.8%).

### Procedure

The current study is based on a general database that has been used for other publications (e.g., Silva et al., 2021) and which was collected from an online platform “FormsUA” - associated to LimeSurvey software, during 32 days, from February to March 2021. Regarding the education format during the data collection period (i.e. COVID-19), participants were still attending classes. To obtain a large sample size, several recruitment strategies were used, including institutional emails and dissemination through digital social networks (i.e., Facebook and Instagram).

Firstly, informed consent was presented with a detailed description of the study, the inclusion criteria (i.e., being Portuguese, studying at a higher education institution, and being at least 18 years old, excluding participants who do not meet the inclusion criteria) and the email addresses of the responsible team for further research details if needed.

The study was approved by the Ethics and Deontology Committee of the University where the research was carried out (n.° 35/2019).

### Measures

#### Sociodemographic Variables

For the current study, information on sex, age, marital status, degree, field of study and student status was collected. For details, cf. Silva et al. (2021).

#### Hospital Anxiety and Depression Scale

The Hospital Anxiety and Depression Scale (HADS) is a self-assessment scale designed to evaluate states of depression and anxiety in clinical and non-clinical settings (Zigmond & Snaith, 1983). The Portuguese version of the HADS (Pais-Ribeiro et al., 2007) has been shown to be reliable and valid. It comprises a Likert type scale with 4 response options (from 0 to 3 points), according to 14 statements: 7 related to anxiety and 7 related to depression. According to the cut-off scores, for each of the scales, from 0 to 7 is considered “*normal*”, from 8 to 10 “*mild*”, from 11 to 14 “*moderate*”, and from 15 to 21 “*severe*”. In the current sample, the Cronbach’s alpha was .84 for anxiety and .77 for depression. Cronbach’s α value for total HADS score was .87.

#### GSES

The GSES assesses the level of effort to sleep (Broomfield & Espie, 2005). The initial Portuguese version of the GSES (Meia-via et al., 2015) demonstrated adequate reliability and validity. This scale has 7 items that evaluate the effort to sleep, presenting statements related to effortful sleep practices, with 0 corresponding to “*not at all*”, 1 “*to some extent*”, and 2 “*very much*”. Scores on this scale range from 0 to 14. In the current study, the Cronbach’s alpha was .77.

#### Statistical Analysis

All statistical analyses were performed using IBM SPSS Statistics version 26.0. Frequencies and percentages were computed for sociodemographic data, and descriptive statistics such as means and standard deviations to characterize the measures used in the study. Pearson’s correlations were calculated to examine possible associations between sleep effort, anxiety and depression. Sex, age, and marital status were included as covariates in some mediation analyses. These variables were controlled because studies indicate that women are more likely to suffer from insomnia (Buysse et al., 2008; Klink, 1992; Morin et al., 2006; Ohayon, 1996, 2002) as well as older adults (Buysse et al., 2008; Lindberg et al., 1997; Morrison et al., 1992; Ohayon, 1996; Olson, 1996; Weissman et al., 1997). Moreover, several studies indicate that single, divorced or widowed individuals are more likely to experience insomnia (Doi et al., 2000; Kawata et al., 2020).

Mediation analysis is a simple method that extracts information about the hypothetical causal mechanisms (Preacher, 2015) and attempts to understand if a mediating variable (*M*) has any influence on the strength of the relationship between two variables: a predictor variable (*X*) and a criterion variable (*Y*). Furthermore, it can explain possible mechanisms on how a variable X has an impact on a variable Y. It is possible to describe this phenomenon as partial mediation when this effect decreases (but not suppresses) the strength of the relationship between X and Y (Baron & Kenny, 1986; Hayes, 2009). Albeit the limitations concerning the mediation analysis in cross-sectional designs, several studies have been used it in behavioral sciences (Shrout & Bolger, 2002).

For the mediation analyses, PROCESS macro v.4.0 (Model 4) for SPSS developed by Hayes (2018) was used, enabling the computation of bootstrapping – a robust and accurate method for estimating the mediated effect (Cheung, 2007) – and the analysis of the indirect effect through a bootstrapping analysis with 5000 re-samples in 95% confidence intervals (Kim & Suh, 2017). Effect size was calculated (Kelley & Preacher, 2012) and were classified as small (*r* = 0.1), moderate (*r* = 0.3), or large (*r* = 0.5) (Cohen, 1992). In the present study, the mediated effect proportion was calculated in two steps. In the first step, the direct effect coefficient was divided by the total effect coefficient. To find the mediated effect as a percentage, the previously calculated value was subtracted from one and multiplied by 100. To perform a mediation analysis, four conditions should be met: the predictor variable should significantly predict the dependent variable; the predictor variable should significantly predict the mediating variable; the mediating variable should significantly predict the dependent variable; and finally, the relationship between the predictor and dependent variable should be reduced after the insertion of the mediating variable (Fairchild & McDaniel, 2017).

## Results

### Descriptive Statistics and Correlations

Descriptive statistics for the assessment instruments were: GSES (*M* = 4.87; *SD* = 0.08; *Min* = 0; *Max* = 14), HADS-A (*M* = 8.39; *SD* = 0.10; *Min* = 0; *Max* = 21), and HADS-D (*M* = 5.44; *SD* = 0.08; *Min* = 0; *Max* = 18).

Considering cut-off points of the HADS, 45.6% of the participants had a “normal” level of anxiety, 22.5% mild, 22.2% moderate, and 9.7% severe anxiety. In terms of depression, 71.4% of the participants had ‘‘normal’’ scores, 19% were mild, 8.8% were moderate, and 0.9% were in the severe depression category.

Regarding correlations between the GSES, HADS-A and HADS-D scales, the statistical tests showed significant associations between all variables with moderate to large effect sizes (Cohen, 1988). The correlation between the GSES and the HADS-A was *r* = .52, whereas the correlation between the GSES and the HADS-D was *r* = .43. Finally, as to the correlation between HADS-A and HADS-D was *r* = .61. The significance for all computed correlations was *p* < .01 (cf. Table 1).

**Table 1.** Means, Standard Deviations and Correlations between GSES, HADS-A, and HADS-D.

	*M*	*SD*	GSES	HADS-A	HADS-D
GSES	4.87	.08	—	.52^*^	.43^*^
HADS-A	8.39	.10		—	.61^*^
HADS-D	5.44	.08			—

Note: M = Mean; *SD* = Standard deviation.

*p < .01.

### Mediation Analyses

#### Sleep Effort as a Mediator in the Effect of Depression on Anxiety

**Figure 1.**
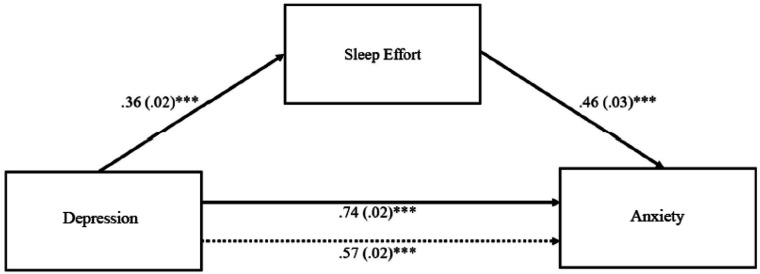
Model with sleep effort as a mediator in the effect of depression on anxiety. *Note*. Unstandardized coefficients are presented with standard errors in parentheses. Dotted line represents direct effect. The indirect effect of depression on anxiety through sleep effort was significant (95% *CI* [.14, .19]). ***p < .001.

The indirect effect of sleep effort in the association between depression and anxiety was significant since the confidence interval at 95% did not pass through the zero value (*b* = 0.16; 95% *BCa CI* [0.14, 0.19]), meaning that with the introduction of the mediator variable “sleep effort” in the model, the effect of depression on anxiety remained significant (partial mediation). Sleep effort mediated approximately 23% of the relationship between depression and anxiety (cf. Figure 1).

#### Sleep Effort as a Mediator in the Effect of Anxiety on Depression

**Figure 2.**
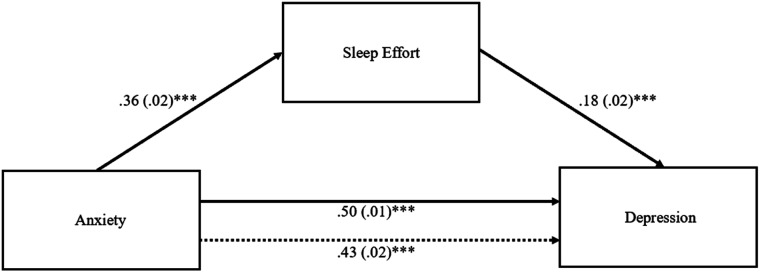
Model with sleep effort as a mediator in the effect of anxiety on depression. *Note*. Unstandardized coefficients are presented with standard errors in parentheses. Dotted line represents direct effect. The indirect effect of anxiety on depression through sleep effort was significant (95% *CI* [.05, .08]). ***p < .001.

The indirect effect of sleep effort in the association between anxiety and depression was significant (*b* = 0.06; 95% *BCa CI* [0.05, 0.08]), meaning that with the introduction of the mediator variable “sleep effort” in the model, the effect of anxiety on depression remained significant (partial mediation). Sleep effort mediated approximately 14% of the relationship between anxiety and depression (cf. Figure 2).

#### Effects of Sleep Effort on Depression and Anxiety Considering Sex, Age and Marital Status as Covariates

After introducing sex, age and marital status as covariates, the indirect effect of sleep effort in the association between depression and anxiety was still significant (*b* = 0.17; 95% *BCa CI* [0.14, 0.19]), meaning that with the introduction of the mediator variable “sleep effort” in the model, the effect of depression on anxiety remained significant (partial mediation). Sleep effort mediated approximately 22% of the relationship between depression and anxiety. The same was observed for the effect of sleep effort in the association between anxiety and depression (*b* = 0.06; 95% *BCa CI* [0.04, 0.08]). Sleep effort mediated approximately 12% of the relationship between anxiety and depression (cf. Table 2).

**Table 2.** Effects of Sleep Effort on Depression and Anxiety Considering the Covariates Sex, Age and Marital Status.

Model	Coefficient	*SE*	Boot LLCI	Boot ULCI
Covariates: Sex, Age and Marital Status				
Dependent Variable: HADS-D				
Total effect of HADS-A on HADS-D	.51	.02	.48	.54
Direct effect of HADS-A on HADS-D	.45	.02	.42	.49
Indirect effect of HADS-A on HADS-D	.06	.01	.04	.08
Dependent variable: HADS-A				
Total effect of HADS-D on HADS-A	.73	.02	.69	.77
Direct effect of HADS-D on HADS-A	.57	.02	.52	.61
Indirect effect of HADS-D on HADS-A	.17	.01	.14	.19

## Discussion

It is known that depression, anxiety, and insomnia are bidirectionally related and are commonly reported comorbid conditions. However, sleep effort is still an under-researched construct. It involves cognitive and behavioral factors that underlie insomniaʼs maintenance and perpetuation (Broomfield & Espie, 2005). This study aims to investigate whether sleep effort mediates anxiety and depression, and vice versa. As a result, and based on findings of the current study, it seems that sleep effort is associated with both anxiety and depression. Further, sleep effort also appears to be a bidirectional mediator between anxiety and depression.

Research has shown that insomnia and anxiety influence one another over time, showing a bidirectional relationship (Glidewell et al., 2015; Jansson-Fröjmark & Lindblom, 2008). Moreover, Batterham et al. (2012) indicated that insomnia precedes depression and increases the risk for a depressive episode. In another study, Jansson-Fröjmark and Lindblom (2008) demonstrated a bidirectional relationship between anxiety, depression, and insomnia over the course of 1 year. This is consistent with the findings of the present study, which found moderate correlations between anxiety, depression and sleep effort.

### Mediation Model

It is interesting to note that, in general, sleep effort, which is a construct quite related to insomnia, seems to have a stronger effect when depression predicts anxiety compared to when anxiety predicts depression. According to our results, sleep effort explained 22% of the relationship between depression and anxiety. There is evidence that insomnia predicts depression and anxiety (Baglioni et al., 2011; Harvey, 2002), and the same is true for sleep effort. Sleep effort is a construct that underlies the maintenance and perpetuation of insomnia (Broomfield & Espie, 2005) and studies indicate that depression can develop over time (Brown et al., 2001); sleep effort it is also related to anxiety, in that many of the symptoms of worry and ruminations are interconnected with each other, as indicated by many of the models of insomnia such as the Cognitive Model of Insomnia (Harvey 2002). This perspective outlines the importance of cognitive processes in insomnia. These processes involve worry, selective attention and monitoring, maladaptive beliefs, and dysfunctional behaviors (Marques et al., 2015). According to this model, excessive worry and ruminations generate hyperarousal and distress, which leads to sleep difficulties (Harvey, 2002). Ballesio et al. (2021) and Perlis et al. (1997) also assert that worries and ruminations are longitudinal precursors to depression and anxiety and are present in insomnia.

A study by Jansson and Linton (2006) aimed to investigate the associations between depression, anxiety and insomnia, and the role of anxiety and depression in the development of insomnia. The study demonstrated that anxiety and depression have a moderate to strong association with insomnia. In addition, they showed that anxiety conferred a higher risk for insomnia than depression. Complementarily, a study by Ellis et al. (2014) showed the importance of insomnia to development of depression. Regarding the association between anxiety and insomnia, and according to Spielman’s (1987) behavioral perspective on insomnia, also called the 3P’s Model (predisposing, precipitating, and perpetuating), anxiety is a broader precipitant of insomnia compared to depression; the former may be present as a predisposing factor (characteristics that increases the vulnerability to insomnia); as a precipitating factor (when it is associated with medical, environmental or psychological factors that trigger insomnia); and as a perpetuating factor (fear of sleeplessness, excessive worries about daytime consequences) (Ellis et al., 2021; Marques et al., 2015; Perlis et al., 2017; Spielman, 1987). Therefore, there is a strong connection between all these factors and anxiety (Buckner et al., 2008).

The present research focused on sleep effort as one facet of insomnia. However, one should note that despite this high association they are different constructs.

### Mediation Model with Covariates

In the mediation analysis performed with sex, age, and marital status as covariates, it was found that there were no changes in comparison to the mediation analysis without these variables. Therefore, even when these variables are controlled for, the effects remain the same.

### Practical Implications

In light of the correlation between sleep effort, anxiety and depression, sleep health and hygiene will have to be taken into account as a preventive measure. Sleep health targets physical, mental, and psychosocial wellness, and there are several dimensions of sleep that influence health, such as: (i) duration of sleep; (ii) consistency of sleep; (iii) timing; (iv) alertness; and (v) satisfaction with sleep (Buysse, 2014). In addition, sleep health is crucial for an effective cognitive functioning, mood, mental health, among others (Watson et al., 2015). The individual whose sleep health is effective will be less likely to strain to sleep. In the same way, sleep hygiene education is crucial in addition to preventive measures, since studies show that better sleep hygiene decreases the risk of insomnia (Gellis et al., 2014). Sleep hygiene refers to a set of behaviors, environmental conditions, and other factors that can be adapted (Stepanski & Wyatt, 2003). According to a study by Baroni et al. (2017), depression and anxiety symptoms decrease when individuals keep sleep healthy behaviors. Thus, all these health-promoting and preventive behaviors bring benefits pertaining to the emergence of sleep problems that can later lead to sleep effort associated with anxiety and depression.

### Limitations

The main limitations of the present study is the cross-sectional design, since it is not a longitudinal study thus it does not allow a discussion on the direction of causality in the associations found (Maxwell & Cole, 2007). The current study examines a sample of Portuguese university students, and it does not account for variables such as culture, somewhat limiting generalizability. This is pertinent since several studies have demonstrated differences between cultures (Blazer et al., 1995). Furthermore, the study data was obtained during the beginning of the COVID-19 pandemic, and this may have affected the results in terms of overall levels of anxiety, depression and insomnia. Literature indicates that the COVID-19 pandemic has had a great impact at all levels, including the physical and mental well-being of individuals (Morin et al., 2021). Adding the fact that several meta-analyses reported rising levels of anxiety, insomnia, and depression during the pandemic (Jahrami et al., 2021; Liu et al., 2021). That said, the present study did not specifically aim to examine the clinically relevant levels of each variable, rather their interrelatedness.

Future studies should include clinical samples and compare results between groups of clinical and non-clinical samples. Additionally, longitudinal studies with sleep effort as a mediating variable should be considered.

## Conclusion

In conclusion, it appears that anxiety and depression are mediated by sleep effort. Sleep effort is one of the many factors that underlie the maintenance of insomnia and precedes other mental disorders such as anxiety and depression. In turn, anxiety and depression may increase the likelihood that an individual will experience insomnia (Ohayon & Roth, 2003). The examination of their relationship, if any, is important for an effective and efficient treatment. In this sense, it may be beneficial in preventing the development of anxiety, depression and insomnia (Ford & Kamerow, 1989; Mellinger et al., 1985). Sleep effort should be considered as a target for interventions aimed at reducing anxiety and depression.
